# Trends and socioeconomic factors associated with low available food variety among Peruvian households: 2015–2024

**DOI:** 10.3389/fnut.2026.1929877

**Published:** 2026-09-18

**Authors:** Miguel A. Arce-Huamani, Blanca M. Sifuentes-Rosario, Maria A. Huanca-Ilaquijo, Candy N. Pacheco-Huarhuachi, Dyego Leandro Bezerra de Souza, Gabriel De la Cruz-Ku, J. Smith Torres-Roman, Luis M. Tasayco-Marquez

**Affiliations:** 1 Research for Evidence, Care, and Public Health, Programa Academico de Medicina Humana, Facultad de Ciencias de la Salud, Universidad Privada Norbert Wiener, Lima, Peru; 2 Graduate Program in Public Health, Federal University of Rio Grande do Norte (UFRN), Natal, RN, Brazil; 3 Universidad Cientifica del Sur, Lima, Peru; 4 Escuela de Posgrado, Universidad Continental, Lima, Peru; 5 Escuela de Posgrado, Universidad Tecnologica del Peru, Lima, Peru

**Keywords:** food availability, food variety, household food expenditure, SDG 2: Zero hunger, socioeconomic inequality

## Abstract

**Background:**

Household food-product variety reflects the breadth of foods reported as available to households but does not directly measure dietary diversity, diet quality, nutrient adequacy, or food insecurity. We examined national trends in low available food variety among Peruvian households from 2015 to 2024 and associated socioeconomic and territorial factors.

**Methods:**

We conducted a repeated cross-sectional analysis of nationally representative ENAHO data from 2015–2024. Low available food variety was defined as ≤71 distinct food products at the household level, corresponding to the lower pooled tertile of the survey-weighted distribution. Survey-weighted prevalence estimates and prevalence ratios were obtained using modified Poisson regression with robust variance, accounting for the complex survey design.

**Results:**

The analysis included 340,296 households, of which 34.2% met the study-specific definition of low available food variety. Prevalence decreased from 33.8% in 2015–2019 to 29.5% in 2020–2021, then increased to 38.0% in 2022–2024, reaching 40.1% in 2024. Compared with 2015–2019, prevalence was higher in 2022–2024 (aPR: 1.28; 95% CI: 1.26–1.30). Higher adjusted prevalence was also observed among households in the Rainforest region, those living in extreme poverty, and those in the lowest tertile of per-capita food expenditure.

**Conclusion:**

Low available food variety increased after 2021 and showed marked socioeconomic and territorial inequalities. These findings reflect differences in household food-product availability and should not be interpreted as direct evidence of dietary diversity, diet quality, nutrient adequacy, or food insecurity.

## 1 Introduction

In this study, available food variety refers to the number of distinct food products reported as available at the household level. This product-count indicator is conceptually distinct from dietary diversity, diet quality, nutrient adequacy, and food insecurity and should not be interpreted as a direct measure of any of these constructs (1). In Latin America and the Caribbean, food insecurity reached 75.7% among adults surveyed during the first pandemic wave (2), while a 13-country analysis estimated that 40.9% of the regional population experienced food insecurity during the same period (3). A multicountry study in Latin America later reported 29.3% mild, 9.2% moderate, and 3.3% severe food insecurity, showing that food constraints persisted beyond the initial emergency phase (4). In South America, moderate food insecurity increased from 4.5% before the pandemic to 16.9% during the pandemic, and severe food insecurity rose from 0.6 to 2.7%, with Peru showing the largest absolute increase (5). These findings provide relevant context for examining household food-product availability across periods of socioeconomic disruption; however, the product-count indicator used in the present study should not be interpreted as a direct measure of food insecurity, dietary diversity, or diet quality.

Food variety and dietary diversity can be operationalized in different ways, and these measures should not be treated as interchangeable. Conventional household dietary diversity measures, such as the Household Dietary Diversity Score (HDDS), generally count the number of predefined food groups consumed by household members over a specified recall period, whereas food variety scores may count individual food items rather than food groups (6, 7). Recent methodological reviews have emphasized substantial heterogeneity in the terminology, scoring approaches, food classifications, and reference periods used to assess dietary diversity and food variety (7, 8). In the present study, the ENAHO-based indicator differs from these established dietary diversity measures because it counts distinct food products reported as available through the household food acquisition/consumption module rather than food groups consumed during a standardized dietary recall. We therefore refer to the outcome specifically as available food variety and do not interpret it as a validated HDDS, dietary diversity score, or direct measure of dietary quality or nutrient adequacy.

Peruvian evidence consistently shows that food insecurity is socially patterned rather than evenly distributed across households. Curi-Quinto et al. (9) reported food insecurity in 24.0% of young Peruvians in 2020, with prevalence reaching 31.7% among participants in the lowest wealth tertile compared with 17.1% among those in the highest wealth tertile. In low-income peri-urban households, Pradeilles et al. (10, 11) showed that food insecurity persisted across different stages of the COVID-19 pandemic and was closely related to income loss, household composition, and food assistance. Similar socioeconomic vulnerability was reported by Zila-Velasque et al. (12), who found a higher prevalence of food insecurity among adults affected by job loss in high-altitude Peruvian cities. Taken together, these studies indicate that household resources and economic shocks are important determinants of food access in Peru, while also highlighting that most previous evidence has relied on experiential food-insecurity measures rather than household-level food-product counts.

Peruvian evidence further indicates that household food access is patterned by socioeconomic and household characteristics. Santos et al. (13) reported higher food insecurity among women-headed households in Villa El Salvador, while Lozada-Urbano et al. (14) showed that poverty and household structure were associated with food-away-from-home expenditure in ENAHO. Hernández-Vásquez et al. (15) also documented a substantial burden of food insecurity among Venezuelan migrant and refugee households in Peru. Together, these findings suggest that socioeconomic position and household composition are relevant to household food access, although previous studies have primarily relied on food-insecurity or expenditure-based outcomes rather than household-level food-product counts.

At the global level, Laborde et al. (16) reported that the unaffordability of healthy and nutrient-adequate diets increased during the COVID-19 pandemic. In Peru, however, nationally representative evidence on long-term changes in household food-product variety remains limited, particularly when using a consistent operational definition across repeated ENAHO rounds. The present study addresses this gap by examining trends from 2015 to 2024 and assessing socioeconomic and territorial inequalities using a harmonized household-level measure of available food variety.

Therefore, the present study aimed to estimate national trends in low available food variety among Peruvian households from 2015 to 2024 and to identify the socioeconomic and territorial factors associated with this condition.

## 2 Materials and methods

### 2.1 Study design and setting

We conducted a repeated cross-sectional analysis using secondary microdata from the Peruvian National Household Survey on Living Conditions and Poverty (ENAHO), collected annually from 2015 to 2024 by the National Institute of Statistics and Informatics of Peru (INEI). Each ENAHO round constitutes an independent, nationally representative household survey; therefore, all time-trend estimates reflect population-level changes across calendar years rather than within-household longitudinal changes.

ENAHO uses a probabilistic, stratified, multistage, and clustered sampling design, with representativeness at national, urban/rural, natural-region, and departmental levels. Primary sampling units correspond to clusters of dwellings or census areas, and households constitute the final sampling units. ENAHO data are generally collected through standardized face-to-face interviews by trained fieldworkers using structured questionnaires and official INEI protocols. During the second quarter of 2020, however, data collection was temporarily conducted by telephone using an abbreviated questionnaire because of COVID-19 restrictions. Face-to-face fieldwork resumed progressively from August 2020, and household visits using the complete questionnaire were again conducted nationwide from October to December 2020 (17).

The present study focused on Peruvian households with complete information on food acquisition/availability, household expenditure, and the covariates required for the analysis. The unit of analysis was the household. We pooled harmonized annual household-level files from 2015 through 2024 and applied the complex survey design in descriptive and inferential analyses using the ENAHO household expansion factor, strata, and clustered primary sampling units. The study was reported according to the Strengthening the Reporting of Observational Studies in Epidemiology (STROBE) guidelines.

### 2.2 Population and sample

The target population comprised private Peruvian households included in ENAHO between 2015 and 2024. For each year, we used the household food acquisition/consumption module and linked it with household, dwelling, socioeconomic, and demographic modules using the household identifiers provided by INEI.

Annual files were harmonized using a common variable structure and consistent household identifiers across survey years. Unique households were identified using year, cluster, dwelling, and household identifiers. Across 2015–2024, the annual household master/SUMARIA files contained 345,109 unique households. Of these, 341,588 were successfully linked to the food acquisition/consumption Module 601 using the same household identifiers, whereas 3,521 households, all from 2022, could not be validly linked and were excluded. Among households linked to Module 601, 1,292 did not have a constructible available food variety score because no valid food-product record remained after applying the food availability/acquisition criteria. No households were excluded because of missing survey-design variables. The final descriptive analytic sample therefore comprised 340,296 households (Figure 1).

**Figure 1:**
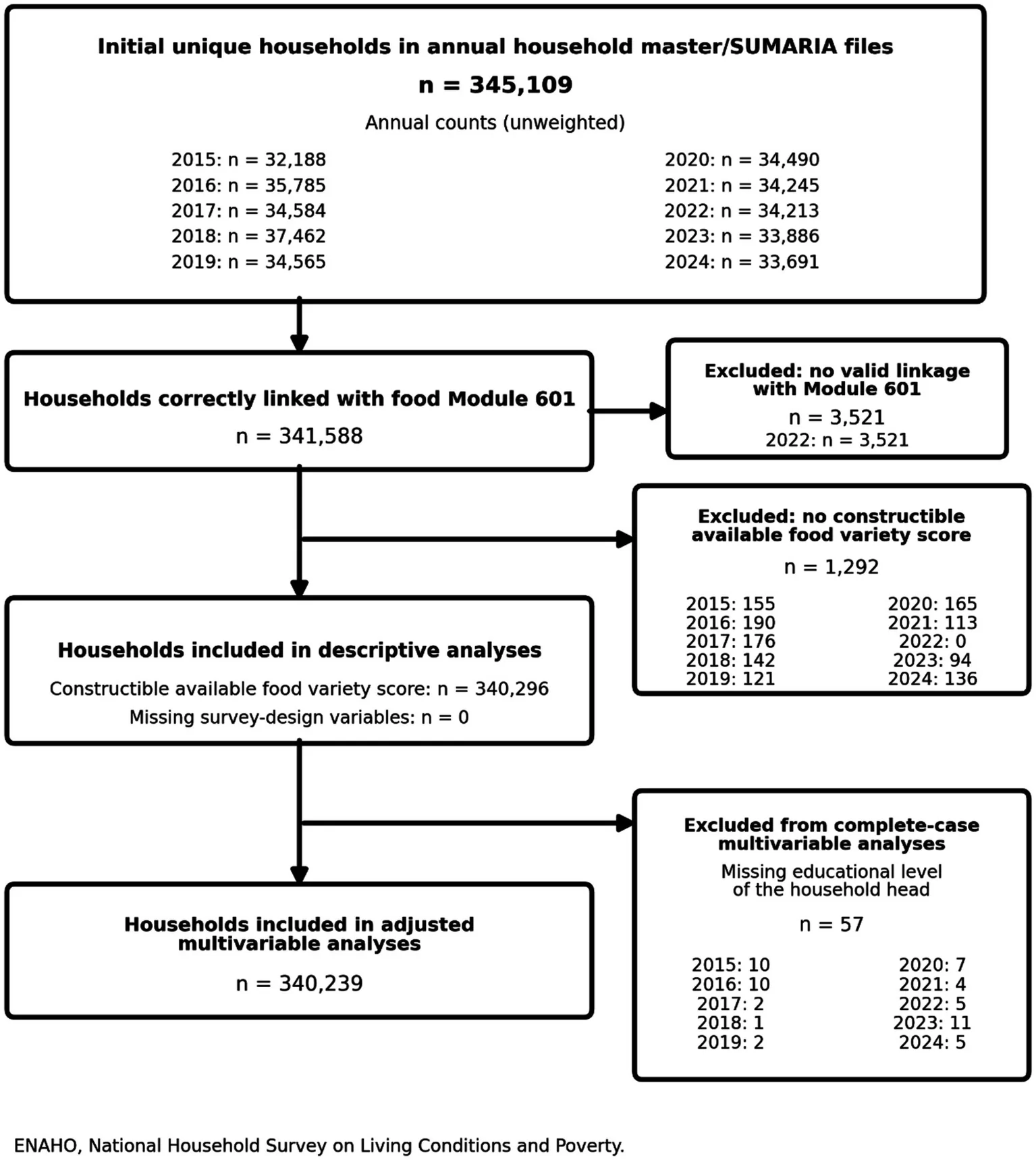
Flow diagram of household selection for the ENAHO 2015–2024 analyses. The annual household master/SUMARIA files contained 345,109 unique households. Of these, 3,521 households, all from 2022, could not be linked to food Module 601, and 1,292 linked households did not have a constructible available food variety score, yielding 340,296 households for descriptive analyses. No households were excluded because of missing survey-design variables. For complete-case multivariable analyses, 57 households with missing educational level of the household head were excluded, leaving 340,239 households in the adjusted models.

The annual descriptive samples were 32,033 households in 2015, 35,595 in 2016, 34,408 in 2017, 37,320 in 2018, 34,444 in 2019, 34,325 in 2020, 34,132 in 2021, 30,692 in 2022, 33,792 in 2023, and 33,555 in 2024. The only missing covariate required for the adjusted analyses was educational level of the household head, which was missing for 57 households: 10 in 2015, 10 in 2016, 2 in 2017, 1 in 2018, 2 in 2019, 7 in 2020, 4 in 2021, 5 in 2022, 11 in 2023, and 5 in 2024. These households were retained in the descriptive analyses but excluded from complete-case multivariable analyses, resulting in 340,239 households in the adjusted models. For temporal analyses, survey years were grouped into three periods: 2015–2019, 2020–2021, and 2022–2024, representing the pre-pandemic, pandemic-disruption, and post-2021 periods, respectively.

### 2.3 Data collection

ENAHO data were collected by INEI through standardized household interviews conducted by trained survey personnel. The survey collects information on household composition, dwelling characteristics, education, employment, income, expenditure, poverty, access to services, and detailed household consumption and expenditure.

The ENAHO food acquisition/consumption Module 601 uses a 15-day reference period and is organized into 50 predefined product rubrics rather than an unrestricted food inventory. Most rubrics refer to foods and beverages commonly obtained for household consumption, including cereals and breads, dairy products, meats, fish and seafood, fats, legumes, vegetables, fruits, beverages, and prepared foods. Several rubrics are intentionally broad or residual, including other dairy products, other vegetables and legumes, other fruits, and other foods for household consumption. Thus, respondents report whether household members obtained, consumed, purchased, or received products within these predefined rubrics. The official module documentation does not describe a separate unrestricted open-ended field for adding arbitrary food items; products not explicitly named are instead accommodated within the corresponding residual “other” categories.

For this study, the ENAHO food acquisition/consumption module was processed at the product level. Food products were identified using the product codes provided by INEI and linked to households using the corresponding household identifiers. A product was considered available when it was recorded as acquired, obtained, or consumed by the household through any of the sources captured in the module, including purchases, self-consumption, self-supply, in-kind acquisition, donations, social programs, or other reported sources. All acquisition sources contributed equally to the food variety score. Each distinct product code was counted only once per household, regardless of the quantity acquired, frequency of acquisition, monetary value, or number of acquisition sources reported for that product. The harmonized analytic files therefore contained one record per household–product combination before aggregation. Product-level records were subsequently aggregated to the household level and linked to the household, dwelling, socioeconomic, demographic, and expenditure files using the household identifiers provided by INEI.

Food expenditure variables were analyzed in annualized nominal Peruvian soles, as recorded in the ENAHO-derived expenditure variables. We did not apply an external inflation deflator because monetary expenditure was not used to estimate changes in real purchasing power or the real cost of food over time; rather, it was included as a descriptive household expenditure indicator and as a socioeconomic covariate in the adjusted analyses. This approach is consistent with previous repeated cross-sectional analyses of ENAHO that have operationalized expenditure amounts in nominal Peruvian soles (18). Nevertheless, nominal expenditure values are affected by changes in the general price level and therefore should not be interpreted as inflation-adjusted temporal changes in purchasing power.

### 2.4 Variables

The primary outcome was low available food variety at the household level. The available food variety score was calculated as the total number of unique food-product codes meeting the availability criterion described above for each household. Thus, each eligible product contributed one unit to the score, irrespective of quantity, expenditure, or acquisition source. The score captures the breadth of distinct food products reported as available to the household and does not represent a validated household dietary diversity score or a measure of individual dietary intake, diet quality, nutrient adequacy, or food insecurity.

Low available food variety was defined using a study-specific fixed threshold derived from the pooled 2015–2024 distribution. Households with ≤71 distinct food products, corresponding to the lower pooled tertile of the survey-weighted available food variety distribution, were classified as having low available food variety. This threshold was not intended to represent a clinically or nutritionally validated cut-off; rather, it was used as a relative, distribution-based criterion to identify households in the lower segment of food-product availability while maintaining the same definition across survey years. Because the threshold is sample-dependent, additional sensitivity analyses were conducted using a stricter cut-off and the continuous food variety score.

The main exposure was survey year, analyzed both as an annual variable from 2015 to 2024 and as a three-level survey period variable: 2015–2019, 2020–2021, and 2022–2024.

Food expenditure was analyzed using three indicators. First, annual household food expenditure was estimated from the food expenditure variables available in ENAHO. Second, annual per-capita household food expenditure was calculated by dividing annual household food expenditure by household size. Third, food expenditure burden was calculated as the proportion of total household expenditure allocated to food. High food expenditure burden was defined as food expenditure representing at least 50% of total household expenditure.

Variables examined in the descriptive and bivariate analyses were selected based on their socioeconomic, territorial, and household-composition relevance. These included area of residence, natural region, department, poverty status, household size, presence of children younger than five years, presence of adults aged 60 years or older, sex of the household head, age of the household head, educational level of the household head, and tertiles of annual per-capita household food expenditure. Presence of adults aged 60 years or older was evaluated descriptively and bivariately but was not included in the multivariable models; the adjusted specification used age of the household head as the age-related covariate and household size as the household-composition covariate, thereby avoiding redundant representation of these household characteristics.

Area of residence was classified as urban or rural. Natural region was categorized as Coast, Highlands, Rainforest, and Metropolitan Lima. Poverty status was classified according to the official ENAHO poverty variable as non-poor, non-extreme poor, or extreme poor. Household size was categorized as 1–2, 3–4, and ≥5 members. Age of the household head was categorized as 18–39, 40–59, and ≥60 years. Educational level of the household head was grouped into no formal/initial education, primary, secondary, and higher education. Detailed operational definitions, original ENAHO variables, recoding procedures, and reference categories are provided in Supplementary Table S1.

### 2.5 Data analysis

All analyses accounted for the ENAHO complex survey design. Household expansion weights were used to obtain nationally representative estimates. For pooled analyses, survey design identifiers were harmonized across years, and year-specific survey strata and primary sampling units were considered to avoid overlap of design identifiers across annual survey rounds.

Descriptive analyses were first performed to characterize the study population. Absolute frequencies were reported as unweighted counts, while percentages were weighted using the ENAHO household expansion factor. Continuous indicators, including annual per-capita food expenditure, food expenditure share, and available food variety score, were summarized using weighted means, medians, and interquartile ranges.

Annual trends were estimated for low available food variety, high food expenditure burden, mean annual per-capita household food expenditure, and mean available food variety score. Survey-weighted 95% confidence intervals were calculated for the annual prevalence estimates of low available food variety and are displayed as error bars in Figure 2. The main temporal interpretation emphasized the three analytical periods because the observed pattern was not linear across 2015–2024. Annual relative change in low available food variety was estimated using a log-linear model with survey year as a continuous predictor. This estimate was interpreted as annual relative change and was not described as an average annual percent change from Joinpoint regression because Joinpoint models were not used.

**Figure 2:**
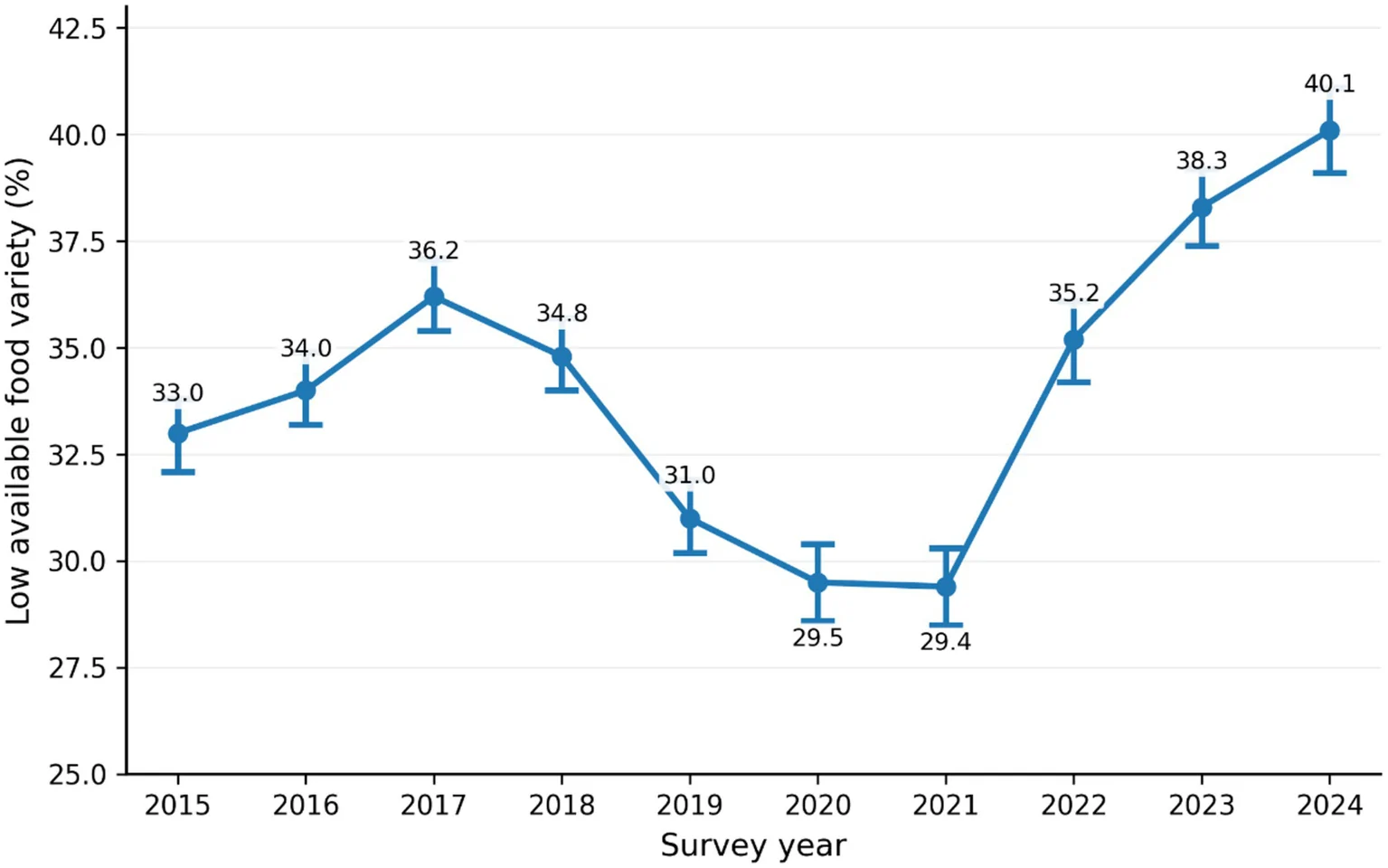
National trend in low available food variety at the household level among Peruvian households, ENAHO 2015–2024. Points represent survey-weighted annual prevalence estimates, and vertical error bars indicate 95% confidence intervals accounting for ENAHO sampling weights, strata, and clustered primary sampling units. Low available food variety was defined using a fixed pooled 2015–2024 threshold of ≤71 distinct food products.

Bivariate associations between low available food variety and each covariate were evaluated using survey-weighted prevalence estimates and crude prevalence ratios. Crude prevalence ratios and 95% confidence intervals were estimated using modified Poisson regression with a log link and robust variance. The bivariate table reported the weighted prevalence of low available food variety, crude prevalence ratio, 95% confidence interval, and *p*-value for each category.

Multivariable analyses were performed using modified Poisson regression with robust variance to estimate adjusted prevalence ratios and 95% confidence intervals. This modeling strategy was selected because the outcome was frequent and odds ratios from logistic regression would overestimate the magnitude of association. Covariates were retained based on conceptual relevance rather than bivariate statistical significance.

Two adjusted models were fitted to distinguish the socioeconomic association of poverty status from the potential contribution of household food expenditure. Model 1 included survey period, area of residence, natural region, poverty status, household size, presence of children younger than five years, sex of the household head, age of the household head, and educational level of the household head. Model 2 additionally included tertiles of annual per-capita household food expenditure. Model 1 was used to evaluate associations before adjustment for food expenditure, whereas Model 2 was considered the fully adjusted model and was used for the main interpretation. This sequential modeling strategy was used because poverty status and food expenditure per capita may represent overlapping socioeconomic pathways.

The adjusted models included area of residence, natural region, and department fixed effects. Natural region was retained as the prespecified territorial indicator of substantive interest, whereas department fixed effects were included to account for residual geographic heterogeneity across departments.

To evaluate whether socioeconomic inequalities changed over time, we tested an interaction between poverty status and survey period. Adjusted predicted prevalence estimates were obtained for each poverty group within each analytical period. Absolute and relative gaps were calculated by comparing extreme-poor and non-poor households within each period.

High food expenditure burden, defined as food expenditure ≥50% of total household expenditure, was analyzed as a secondary outcome. Annual and period-specific weighted estimates were calculated, and a secondary modified Poisson model was used to identify factors associated with high food expenditure burden.

Three sensitivity analyses were performed to assess the robustness of the findings to alternative outcome specifications and potential variation in product coding across survey years. First, low available food variety was redefined using a stricter fixed threshold corresponding to the pooled survey-weighted 25th percentile (≤64 distinct food products), and the adjusted modified Poisson model was refitted. Second, the available food variety score was analyzed as a continuous outcome using survey-weighted linear regression, with adjusted mean differences and 95% confidence intervals estimated using the same covariate structure as the primary model. Third, because the number of observed food-product codes varied across the annual analytic files, we reconstructed the score using only the 463 product codes observed in all ten survey years and repeated the temporal analysis. All three sensitivity analyses used the same covariate structure as Model 2, including department fixed effects, and incorporated the ENAHO sampling weights, strata, and clustered primary sampling units.

Multicollinearity among the substantive covariates included in the fully adjusted model (Model 2) was assessed using variance inflation factors (VIFs), with values below 4 considered acceptable. Department fixed-effect indicators were not included in the VIF summary because they were used to control for residual geographic heterogeneity rather than interpreted as substantive exposure variables. The corresponding VIF estimates are presented in Supplementary Table S2. Statistical significance was assessed using two-sided *p*-values, with *p* < 0.05 considered statistically significant.

## 3 Results

A total of 340,296 Peruvian households from ENAHO 2015–2024 were included in the analysis. Of these, 47.4% corresponded to the 2015–2019 period, 20.7% to 2020–2021, and 31.8% to 2022–2024. Most households had 3–4 members, had no children younger than 5 years, and were headed by men. Regarding socioeconomic characteristics, 80.4% of households were classified as non-poor, 16.3% as non-extreme poor, and 3.3% as extreme poor. Most households were located in urban areas, although a relevant proportion lived in the Highlands and Rainforest regions. Overall, 34.2% of Peruvian households had low available food variety, defined as having ≤71 distinct food products available at the household level (Table 1).

**Table 1:** Characteristics of the study population, Peru, ENAHO 2015–2024.

Characteristic	*N*	%
Total households	340,296	100.0
Survey period		
2015–2019	173,800	47.4
2020–2021	68,457	20.7
2022–2024	98,039	31.8
Household size		
1–2 members	116,640	32.6
3–4 members	136,632	41.6
≥5 members	87,024	25.8
Children younger than 5 years		
No children <5 years	267,605	79.2
At least one child <5 years	72,691	20.8
Adults aged 60 years or older		
No adults ≥60 years	208,293	61.2
At least one adult ≥60 years	132,003	38.8
Sex of household head		
Male	235,418	67.2
Female	104,878	32.8
Age of household head		
18–39 years	76,768	22.5
40–59 years	149,695	44.3
≥60 years	113,833	33.1
Educational level of household head		
No formal/initial	21,124	5.4
Primary	114,554	30.2
Secondary	123,919	39.6
Higher	80,642	24.9
Poverty status		
Non-poor	274,024	80.4
Non-extreme poor	53,328	16.3
Extreme poor	12,944	3.3
Food expenditure per capita		
Lowest tertile	119,980	33.3
Middle tertile	109,477	33.3
Highest tertile	110,839	33.3
Food expenditure burden		
<50% of total household expenditure	259,250	81.0
≥50% of total household expenditure	81,046	19.0
Area of residence		
Urban	234,359	80.9
Rural	105,937	19.1
Natural region		
Coast	101,888	22.7
Highlands	128,430	33.6
Rainforest	69,721	11.8
Metropolitan Lima	40,257	31.9
Main outcome		
Not low available food variety	212,743	65.8
Low available food variety	127,553	34.2

*N* values are unweighted; percentages are weighted using the ENAHO household expansion factor. Missing data were observed for educational level of the household head (n = 57). Low available food variety was defined as households in the lowest pooled tertile of available food variety at the household level, using a fixed cut-off of ≤71 distinct food products across the pooled 2015–2024 sample. This indicator captures the breadth of distinct food products available to the household, not individual dietary intake, nutrient adequacy, diet quality, or food insecurity.

Authors’ analysis of ENAHO 2015–2024.

In the bivariate analysis, low available food variety showed clear socioeconomic and geographic patterning. Prevalence was higher among rural households, households living in the Rainforest and Highlands, and those classified as poor, particularly the extreme poor. A similarly pronounced gradient was observed across per-capita food expenditure tertiles, with the highest prevalence among households in the lowest tertile. Household composition and characteristics of the household head were also associated with the outcome, although these patterns differed across categories (Table 2).

**Table 2:** Bivariate prevalence and crude association with low available food variety among Peruvian households, ENAHO 2015–2024.

Variable	Weighted prevalence of low available food variety (%)	Crude PR	95% CI	*p*-value
Survey period				
2015–2019	33.8	Reference		
2020–2021	29.5	0.87	0.85–0.89	<0.001
2022–2024	38.0	1.12	1.10–1.14	<0.001
Area of residence				
Urban	31.1	Reference		
Rural	47.6	1.53	1.51–1.56	<0.001
Natural region				
Coast	27.4	Reference		
Highlands	40.2	1.47	1.44–1.50	<0.001
Rainforest	44.2	1.61	1.58–1.65	<0.001
Metropolitan Lima	29.1	1.06	1.03–1.09	<0.001
Poverty status				
Non-poor	30.1	Reference		
Non-extreme poor	45.9	1.52	1.50–1.55	<0.001
Extreme poor	78.1	2.60	2.55–2.64	<0.001
Food expenditure per capita				
Highest tertile	20.7	Reference		
Middle tertile	27.0	1.30	1.28–1.33	<0.001
Lowest tertile	55.1	2.67	2.61–2.72	<0.001
High food expenditure burden				
<50%	33.8	Reference		
≥50%	36.1	1.07	1.05–1.09	<0.001
Household size				
1–2 members	54.5	Reference		
3–4 members	26.3	0.48	0.48–0.49	<0.001
≥5 members	21.4	0.39	0.38–0.40	<0.001
Children younger than 5 years				
No children <5 years	36.8	Reference		
At least one child <5 years	24.3	0.66	0.65–0.67	<0.001
Adults aged 60 years or older				
No adults ≥60 years	31.7	Reference		
At least one adult ≥60 years	38.2	1.20	1.19–1.22	<0.001
Sex of household head				
Male	33.8	Reference		
Female	35.1	1.04	1.02–1.05	<0.001
Age of household head				
18–39 years	34.4	Reference		
40–59 years	29.7	0.86	0.85–0.88	<0.001
≥60 years	40.2	1.17	1.15–1.19	<0.001
Educational level of household head				
Higher	28.5	Reference		
Secondary	30.6	1.07	1.05–1.10	<0.001
Primary	40.2	1.41	1.38–1.44	<0.001
No formal/initial	54.7	1.92	1.88–1.97	<0.001

*N* values are unweighted; percentages are weighted using the ENAHO household expansion factor. Crude PRs and 95% CIs were estimated using modified Poisson regression with robust variance and accounted for the ENAHO complex survey design, including sampling weights, strata, and clustered primary sampling units. Low available food variety refers to household-level food-product availability and should not be interpreted as a direct measure of individual dietary intake, diet quality, nutrient adequacy, or food insecurity.

Authors’ analysis of ENAHO 2015–2024.

Table 3 summarizes the distribution of household food expenditure and available food variety. The mean available food variety score was 82.5 products, while food expenditure indicators showed substantial variation across households. Detailed measures of central tendency and dispersion are presented in Table 3.

**Table 3:** Food expenditure and available food variety indicators, Peru, ENAHO 2015–2024.

Indicator	Mean	Median	P25	P75
Annual per-capita household food expenditure, PEN	2702.8	2381.4	1636.4	3382.9
Food expenditure share of total household expenditure	0.4	0.3	0.2	0.5
Available food variety score, number of products	82.5	83.0	64.0	102.0

Food expenditure values are annualized nominal Peruvian soles at the household level. Per-capita values were calculated by dividing annual household food expenditure by household size. Monetary values should not be interpreted as inflation-adjusted changes in purchasing power over time because no external deflator was applied in this analysis. The available food variety score counts distinct food products available at the household level.

Source: Authors’ analysis of ENAHO 2015–2024.

In the multivariable analysis, several factors remained independently associated with low available food variety. Compared with 2015–2019, households surveyed during 2020–2021 had a slightly lower adjusted prevalence, whereas those surveyed during 2022–2024 had a higher adjusted prevalence. Rural residence remained positively associated with low available food variety. Compared with households in the Coast, those in the Highlands, Rainforest, and Metropolitan Lima had higher adjusted prevalence, with the strongest territorial association observed in the Rainforest. Poverty also remained associated with the outcome, particularly among extreme-poor households. Household size showed an inverse association, with households comprising 3–4 members and ≥5 members having lower adjusted prevalence than households with 1–2 members. The presence of children younger than 5 years, female sex of the household head, and older age of the household head were also associated with lower adjusted prevalence. In contrast, households headed by persons with no formal or only initial education had a higher adjusted prevalence than those headed by persons with higher education.

Associations from Model 1 were estimated before adjustment for per-capita food expenditure, whereas the estimates presented in Table 4 correspond to Model 2, the fully adjusted model. Model 1 estimates are presented separately in Supplementary Table S7 to allow assessment of changes after incorporating household food expenditure. In Model 2, the strongest association was observed for food expenditure per capita: households in the lowest tertile had more than three times the adjusted prevalence of low available food variety compared with those in the highest tertile (aPR: 3.37; 95% CI: 3.31–3.43) (Table 4). The adjusted associations between survey period and low available food variety are also summarized separately in Supplementary Table S3.

**Table 4:** Fully adjusted model (Model 2) of factors associated with low available food variety among Peruvian households, ENAHO 2015–2024.

Associated factors	Crude PR (95% CI)	*p*-value	Adjusted PR (95% CI)	*p*-value
Survey period				
2015–2019	Reference		Reference	
2020–2021	0.87 (0.85–0.89)	<0.001	0.93 (0.91–0.95)	<0.001
2022–2024	1.12 (1.10–1.14)	<0.001	1.28 (1.26–1.30)	<0.001
Area of residence				
Urban	Reference		Reference	
Rural	1.53 (1.51–1.56)	<0.001	1.12 (1.11–1.14)	<0.001
Natural region				
Coast	Reference		Reference	
Highlands	1.47 (1.44–1.50)	<0.001	1.18 (1.15–1.21)	<0.001
Rainforest	1.61 (1.58–1.65)	<0.001	1.43 (1.37–1.49)	<0.001
Metropolitan Lima	1.06 (1.03–1.09)	<0.001	1.05 (1.02–1.09)	0.002
Poverty status				
Non-poor	Reference		Reference	
Non-extreme poor	1.52 (1.50–1.55)	<0.001	1.13 (1.12–1.15)	<0.001
Extreme poor	2.60 (2.55–2.64)	<0.001	1.52 (1.48–1.55)	<0.001
Household size				
1–2 members	Reference		Reference	
3–4 members	0.48 (0.48–0.49)	<0.001	0.42 (0.42–0.43)	<0.001
≥5 members	0.39 (0.38–0.40)	<0.001	0.27 (0.27–0.28)	<0.001
Children younger than 5 years				
No children <5 years	Reference		Reference	
At least one child <5 years	0.66 (0.65–0.67)	<0.001	0.89 (0.87–0.90)	<0.001
Sex of household head				
Male	Reference		Reference	
Female	1.04 (1.02–1.05)	<0.001	0.95 (0.93–0.96)	<0.001
Age of household head				
18–39 years	Reference		Reference	
40–59 years	0.86 (0.85–0.88)	<0.001	0.89 (0.87–0.90)	<0.001
≥60 years	1.17 (1.15–1.19)	<0.001	0.88 (0.86–0.89)	<0.001
Educational level of household head				
Higher	Reference		Reference	
Secondary	1.07 (1.05–1.10)	<0.001	0.97 (0.95–0.99)	<0.001
Primary	1.41 (1.38–1.44)	<0.001	1.01 (0.99–1.03)	0.239
No formal/initial	1.92 (1.88–1.97)	<0.001	1.09 (1.06–1.12)	<0.001
Food expenditure per capita				
Highest tertile	Reference		Reference	
Middle tertile	1.30 (1.28–1.33)	<0.001	1.68 (1.65–1.72)	<0.001
Lowest tertile	2.67 (2.61–2.72)	<0.001	3.37 (3.31–3.43)	<0.001

PR, prevalence ratio; aPR, adjusted prevalence ratio; CI, confidence interval. Model 2 was estimated using modified Poisson regression with robust variance and included survey period, area of residence, natural region, poverty status, household size, presence of children younger than five years, sex, age and educational level of the household head, per-capita household food expenditure tertile, and department fixed effects. Model 1 included the same covariates except per-capita household food expenditure and is presented in Supplementary Table S7. Low available food variety refers to household-level food-product availability and should not be interpreted as a direct measure of individual dietary intake, diet quality, nutrient adequacy, or food insecurity. Complete-case analysis was used (*n* = 340,239); 57 households with missing information on educational level of the household head were excluded from the adjusted analyses.

Authors’ analysis of ENAHO 2015–2024.

Annual estimates showed a non-linear temporal pattern. The prevalence of low available food variety was relatively stable between 2015 and 2019, decreased during 2020–2021, and then increased markedly from 2022 onward, reaching 40.1% in 2024. In parallel, the mean available food variety score declined from 85.8 products in 2021 to 78.2 products in 2024. The proportion of households with high food expenditure burden increased during 2020 and remained above pre-pandemic levels during 2021–2024. Mean nominal annual food expenditure per capita increased across the study period, but these values should be interpreted cautiously because no external deflator was applied (Table 5). This temporal pattern is shown in Figure 2, which presents the annual survey-weighted prevalence estimates with their corresponding 95% confidence intervals.

**Table 5:** Annual trends in low available food variety and food expenditure burden, Peru, ENAHO 2015–2024.

Year	Households, n	Low available food variety (%)	High food expenditure burden (%)	Mean annual food expenditure per capita, PEN	Mean food expenditure share (%)	Mean available food variety score
2015	32,033	33.0	17.1	2287.0	34.8	83.8
2016	35,595	34.0	16.1	2360.0	34.1	83.2
2017	34,408	36.2	16.3	2376.5	33.9	81.2
2018	37,320	34.8	15.6	2443.3	33.5	82.3
2019	34,444	31.0	16.0	2538.7	33.7	85.0
2020	34,325	29.5	23.8	2615.4	38.5	85.5
2021	34,132	29.4	21.3	2810.5	37.2	85.8
2022	30,692	35.2	21.2	3040.3	36.8	81.5
2023	33,792	38.3	21.1	3153.7	36.7	79.1
2024	33,555	40.1	20.1	3215.2	36.2	78.2

N values are unweighted; percentages and means are weighted using the ENAHO household expansion factor. Food expenditure values are annualized nominal Peruvian soles and should not be interpreted as inflation-adjusted changes over time.

Authors’ analysis of ENAHO 2015–2024.

Period-specific stratified estimates showed that socioeconomic and territorial inequalities persisted throughout the study period. Low available food variety remained consistently more prevalent among rural than urban households and among households living in poverty, particularly those in extreme poverty (Supplementary Table S4).

Sensitivity analyses supported the main temporal pattern. When low available food variety was redefined using the stricter pooled 25th-percentile threshold (≤64 products), its weighted prevalence was 25.7% in 2015–2019, 21.1% in 2020–2021, and 29.0% in 2022–2024. Compared with 2015–2019, the adjusted prevalence remained lower in 2020–2021 (aPR: 0.89; 95% CI: 0.87–0.91) and higher in 2022–2024 (aPR: 1.30; 95% CI: 1.27–1.32). When the food variety score was analyzed continuously, households surveyed in 2020–2021 had an adjusted mean of 1.04 additional products (95% CI: 0.66–1.43), whereas those surveyed in 2022–2024 had 7.76 fewer products (adjusted mean difference: −7.76; 95% CI: −8.09 to −7.42) than households surveyed in 2015–2019. Restricting the score to the 463 product codes observed in all ten survey years produced virtually unchanged temporal estimates (2020–2021: aPR: 0.93; 95% CI: 0.91–0.95; 2022–2024: aPR: 1.28; 95% CI: 1.26–1.30) (Supplementary Tables S5, S6).

## 4 Discussion

This study found that approximately one in three Peruvian households met the study-specific definition of low available food variety between 2015 and 2024. The prevalence declined temporarily during 2020–2021 and increased after 2021, reaching 40.1% in 2024. Importantly, this outcome represents a household-level count of distinct food products reported as available and should not be interpreted as a direct measure of dietary diversity, diet quality, nutrient adequacy, or food insecurity. Therefore, the temporal pattern indicates changes in the breadth of reported household food-product availability under the operational definition used in this study. Low available food variety was more prevalent among households with lower per-capita food expenditure, those living in poverty, rural households, and households located in the Rainforest and Highlands, indicating marked socioeconomic and territorial patterning of the indicator.

The present study showed that low available food variety affected a substantial proportion of Peruvian households, reflecting a household-level limitation in the range of food products available rather than a direct measure of individual dietary intake or diet quality. This finding is consistent with recent Peruvian and regional evidence showing that food access constraints remain highly prevalent, although direct numerical comparison is limited because most studies used experiential food-insecurity scales rather than product-count indicators. This distinction is also consistent with the broader measurement literature, which shows that food-item counts, food-group diversity scores, and household dietary diversity indicators capture related but methodologically distinct dimensions of food variety and dietary diversity (6–8).

Previous Peruvian studies have consistently documented substantial food insecurity across different population groups, including low-income households, young adults, and residents of geographically vulnerable areas (9–12). Similar socioeconomic gradients have been reported elsewhere in Latin America (3, 19). Although this evidence provides an important context for understanding constraints on household food access, these studies primarily used experiential food-insecurity measures and are therefore not directly comparable with the household product-count indicator used here. Rather than implying equivalence between these constructs, our findings should be interpreted as complementary evidence of socioeconomic patterning in household food-product availability.

The temporal pattern observed in this study was non-linear, with a temporary reduction during 2020–2021 followed by a marked increase in 2022–2024. The reduction during the pandemic period should be interpreted cautiously. During the second quarter of 2020, ENAHO temporarily shifted to telephone interviews using an abbreviated questionnaire, followed by a progressive return to face-to-face data collection (17). Pandemic-related mobility restrictions, changes in household acquisition and reporting practices, and food-assistance responses may therefore have influenced both actual household food availability and its measurement during this period. The subsequent increase after 2021 occurred in a context in which previous studies have linked income losses, higher food prices, and supply-chain disruptions to constraints in food access (20, 21); however, these mechanisms were not directly evaluated in the present study. Because food expenditure was analyzed in nominal Peruvian soles without an inflation deflator, the expenditure trends cannot be interpreted as changes in real purchasing power. Accordingly, the increase observed during 2022–2024 should be interpreted as a change in the prevalence of the study-specific household food-product availability indicator rather than as evidence that inflation or loss of purchasing power caused the observed trend.

The socioeconomic gradient was one of the clearest findings of this study. Households living in poverty and those in the lowest tertile of per-capita food expenditure had a substantially higher prevalence of low available food variety, demonstrating marked socioeconomic patterning of the household food-product availability indicator. This pattern is consistent with Bonilla-Aguilar and Bernabe-Ortiz (1), who found that the nutritional quality of available household foods in Peru was associated with food expenditure and differed by area of residence. Similarly, Curi-Quinto et al. (9) reported higher food insecurity in the lowest wealth tertile than in the highest wealth tertile, while Pradeilles et al. (10) found that reduced income and lower socioeconomic status were associated with greater food insecurity in low-income Peruvian households. Evidence from Brazil also supports the broader socioeconomic pattern: Manfrinato et al. (19) found that moderate or severe food insecurity in favelas was concentrated among households with lower income and lower education. At the regional level, Vasconcelos-Neto et al. (5) showed that low-income households were particularly susceptible to pandemic-related food insecurity in South America. Although these studies provide relevant socioeconomic context, their food-insecurity measures are not directly comparable with the product-count indicator used in the present study. Thus, our findings indicate socioeconomic inequalities in household food-product availability without implying that this indicator directly measures food insecurity, dietary quality, nutrient adequacy, or real purchasing power.

Territorial inequalities were also evident, with rural households and those located in the Highlands and Rainforest showing higher probability of low available food variety than households in the Coast. This pattern is consistent with previous Peruvian evidence. Curi-Quinto et al. (9) found higher food insecurity in rural areas than in urban areas and higher prevalence in the jungle and highlands than on the coast. Zila-Velasque et al. (12) also documented a high burden of food insecurity in high-altitude Peruvian cities, suggesting that geographic location remains relevant even outside strictly rural settings. These territorial gaps may reflect structural barriers in market connectivity, transportation costs, seasonality, access to fresh foods, and long-standing inequalities in the Peruvian food system. Similar territorial gradients have been reported in other Latin American countries. Novoa-Sanzana et al. (4) found that rural residence was associated with food insecurity in Chile, while Giacoman et al. (22) reported greater food-insecurity vulnerability in rural Chilean households during the pandemic. Therefore, the higher burden observed in the Peruvian Highlands and Rainforest should not be interpreted only as a household-level economic problem, but also as a marker of unequal territorial integration into food markets and public infrastructure.

The associations observed for household composition should be interpreted primarily in light of how the outcome was constructed. Larger households and households with children younger than five years had a lower adjusted prevalence of low available food variety; however, these findings should not be interpreted as evidence of better dietary conditions or lower nutritional vulnerability. Because the outcome represents the total number of distinct food products available at the household level, households with more members may accumulate a broader range of products simply because they purchase or obtain foods for individuals with different ages, preferences, or needs. This measurement feature may partly explain the inverse associations observed for household size and the presence of young children. These findings therefore differ conceptually from studies using individual dietary indicators or experiential food-insecurity measures. For example, Novoa-Sanzana et al. (4) reported greater food insecurity among larger households and those with children younger than ten years, while Pradeilles et al. (10) found greater food insecurity among Peruvian households with two or more children younger than five years. Accordingly, the household-composition associations in the present study should be interpreted as differences in the breadth of household-level food-product availability rather than as protective effects on diet quality, nutrient adequacy, or food security.

This study contributes to the literature by providing nationally representative evidence on household-level available food variety in Peru over a ten-year period that includes the pre-pandemic period, the acute pandemic years, and the post-pandemic recovery phase. Unlike studies focused exclusively on experiential food insecurity or individual dietary intake, this analysis captures the diversity of food products available within households and links it to socioeconomic, territorial, and demographic inequalities. The findings are generalizable to Peruvian households represented in ENAHO and may also be informative for other middle-income countries where food availability is mediated by market access, household purchasing power, and territorial infrastructure. However, generalization should be made with caution because the indicator reflects available food variety at the household level and does not measure individual consumption, nutrient adequacy, dietary quality, or intra-household food distribution.

The main strengths of this study include the use of a large nationally representative sample of Peruvian households, the ten-year analytical window, the application of survey weights and complex sampling design, and the estimation of prevalence ratios using robust Poisson models. The use of a fixed pooled cut-off for low available food variety also allowed temporal comparisons across survey years. Nevertheless, several limitations must be acknowledged. First, the repeated cross-sectional design prevents causal inference. Second, available food variety does not capture quantities consumed, nutritional quality, food preparation, ultra-processed food intake, or individual dietary adequacy. Third, food expenditure values were analyzed in nominal Peruvian soles and should not be interpreted as inflation-adjusted purchasing power. Fourth, residual confounding may persist because unmeasured factors such as local food prices, market distance, seasonality, household food preferences, and food assistance were not fully captured.

An additional limitation concerns the comparability of the product-count indicator across survey years. The number of food-product codes observed in the annual analytic files was not identical across years, which could theoretically influence temporal comparisons. However, a sensitivity analysis restricted to the 463 product codes observed in all ten survey years yielded nearly identical temporal associations to those of the primary analysis, suggesting that variation in the observed product-code universe was unlikely to explain the main temporal pattern. Nevertheless, residual differences in product classification, reporting, or survey implementation across years cannot be completely excluded. In addition, because Module 601 is based on predefined product rubrics with residual “other” categories rather than an unrestricted food inventory, the available food variety score is inherently dependent on the structure and coding framework of the survey module. Changes in data collection during the pandemic may also have influenced household reporting and comparability across years.

In conclusion, low available food variety, defined here using a study-specific household-level count of distinct food products, was common among Peruvian households and increased after 2021. Higher prevalence was observed among households characterized by poverty, lower per-capita food expenditure, rural residence, and residence in the Highlands or Rainforest. These findings identify socioeconomic and territorial inequalities in reported household food-product availability but do not directly demonstrate differences in individual dietary intake, dietary diversity, diet quality, nutrient adequacy, or food insecurity. Household food-product variety may therefore be considered a complementary descriptive indicator alongside validated measures of dietary diversity, food insecurity, and food expenditure in future surveillance and research.

## Data Availability

The raw data supporting the conclusions of this article will be made available by the authors, without undue reservation.
