# Supplementary material for: Trends and socioeconomic factors associated with low available food variety among Peruvian households: 2015–2024
Source: Front Nutr. 2026 Sep 18;13:1929877. doi: 10.3389/fnut.2026.1929877 (PMC13630598; doi:10.3389/fnut.2026.1929877)
Supplement: Supplementary file 1 [file Table_1.DOCX]

**Table S1.** Variables used, operational definitions, and recoding rules

| Manuscript variable | Operational definition | Recoding rule | Original variable(s) | Final categories | Reference category |
| --- | --- | --- | --- | --- | --- |
| Low available food variety | Low breadth of distinct food products available at the household level, defined using the study-specific threshold | 1 if ≤71 products; 0 otherwise | n_food_products, Module 601 | No; Yes | No |
| Available food variety score | Count of distinct food products available to the household | Each product counted once per household | prod_code, n_food_products | Continuous count | Not applicable |
| Survey period | Analytical survey period | 2015–2019, 2020–2021, 2022–2024 | anio | Three periods | 2015–2019 |
| Area of residence | Urban/rural household location | Derived from sampling stratum | estrato, area | Urban; Rural | Urban |
| Natural region | Natural macro-region of residence | Derived from survey domain | dominio, region_nat | Coast; Highlands; Rainforest; Metropolitan Lima | Coast |
| Poverty status | Official poverty classification | Original ENAHO categories | poverty_code | Non-poor; Non-extreme poor; Extreme poor | Non-poor |
| Household size | Number of household members | 1–2, 3–4, ≥5 members | hh_size, hh_members | Three categories | 1–2 members |
| Children younger than 5 years | Presence of child aged <5 years | 1 if at least one child <5 | n_under5, has_under5 | No; Yes | No |
| Adults aged 60 years or older | Presence of adult aged ≥60 years | 1 if at least one adult ≥60 | n_older60, has_older60 | No; Yes | No |
| Sex of household head | Sex of household head | Original coding | head_sex | Male; Female | Male |
| Age of household head | Age of household head | 18–39, 40–59, ≥60 years | head_age | Three categories | 18–39 years |
| Educational level of household head | Highest educational level | Collapsed into four groups | head_educ_code, head_educ_level4 | No formal/initial; Primary; Secondary; Higher | Higher |
| Food expenditure per capita | Household food expenditure divided by household size | Pooled weighted tertiles | food_pc_sum | Lowest; Middle; Highest | Highest |
| High food expenditure burden | Food expenditure ≥50% of total household expenditure | 1 if food share ≥0.50 | food_share_total | <50%; ≥50% | <50% |

Note: Available food variety was derived from the ENAHO food acquisition/consumption module at household level. The indicator includes products acquired, obtained, or available to the household through purchase, self-consumption, in-kind acquisition, donations, programs, or other sources recorded in the module. The fixed cut-off of ≤71 products corresponds to the lower pooled tertile of the 2015–2024 distribution.

**Table S2.** Variance inflation factors for substantive covariates included in the fully adjusted model (Model 2)

| Covariate block | Maximum VIF | Mean VIF | Assessment |
| --- | --- | --- | --- |
| Age of household head | 2.64 | 2.51 | Acceptable |
| Area of residence | 1.97 | 1.97 | Acceptable |
| Children younger than 5 years | 1.75 | 1.75 | Acceptable |
| Educational level of household head | 2.88 | 2.25 | Acceptable |
| Food expenditure per capita | 2.85 | 2.47 | Acceptable |
| Household size | 2.49 | 2.37 | Acceptable |
| Natural region | 2.24 | 1.73 | Acceptable |
| Poverty status | 1.59 | 1.40 | Acceptable |
| Sex of household head | 1.54 | 1.54 | Acceptable |
| Survey period | 1.55 | 1.44 | Acceptable |

Note: VIF, variance inflation factor. VIFs were calculated for the substantive covariates included in Model 2; department fixed-effect indicators were not included in the VIF summary. Values below 4 were considered acceptable. No problematic multicollinearity was identified among the evaluated covariates.

**Table S3.** Fully adjusted association (Model 2) between survey period and low available food variety

| Survey period | Adjusted PR (95% CI) | p-value |
| --- | --- | --- |
| 2015–2019 | Reference |  |
| 2020–2021 | 0.93 (0.91–0.95) | <0.001 |
| 2022–2024 | 1.28 (1.26–1.30) | <0.001 |

Note: aPR, adjusted prevalence ratio; CI, confidence interval. Estimates were obtained using modified Poisson regression with robust variance. Model 2 included survey period, area of residence, natural region, poverty status, household size, presence of children younger than five years, sex, age and educational level of the household head, per-capita household food expenditure tertile, and department fixed effects. The analysis incorporated the ENAHO complex survey design. Complete-case analysis was used (n = 340,239).

**Table S4.** Weighted prevalence of low available food variety by period and key strata

| Stratification variable | Period | n | Low available food variety (%) | Mean food expenditure per capita, PEN | Mean food expenditure share (%) |
| --- | --- | --- | --- | --- | --- |
| Area |  |  |  |  |  |
| Urban | 2015–2019 | 116,856 | 29.6 | 2547.4 | 30.5 |
| Rural | 2015–2019 | 56,944 | 50.2 | 1843.2 | 47.7 |
| Urban | 2020–2021 | 47,689 | 27.3 | 2813.6 | 34.6 |
| Rural | 2020–2021 | 20,768 | 39.2 | 2280.6 | 52.4 |
| Urban | 2022–2024 | 69,814 | 35.6 | 3237.6 | 33.6 |
| Rural | 2022–2024 | 28,225 | 49.0 | 2683.1 | 50.5 |
| Poverty status |  |  |  |  |  |
| Non-poor | 2015–2019 | 141,809 | 29.8 | 2642.0 | 31.6 |
| Non-extreme poor | 2015–2019 | 26,224 | 48.4 | 1319.6 | 45.0 |
| Extreme poor | 2015–2019 | 5,767 | 81.7 | 752.6 | 48.9 |
| Non-poor | 2020–2021 | 54,467 | 25.8 | 3065.9 | 35.4 |
| Non-extreme poor | 2020–2021 | 11,435 | 37.9 | 1540.9 | 46.1 |
| Extreme poor | 2020–2021 | 2,555 | 69.4 | 888.0 | 51.4 |
| Non-poor | 2022–2024 | 77,748 | 33.4 | 3551.6 | 33.8 |
| Non-extreme poor | 2022–2024 | 15,669 | 48.2 | 1844.4 | 45.2 |
| Extreme poor | 2022–2024 | 4,622 | 79.2 | 1103.0 | 50.8 |

Note: N values are unweighted; percentages and means are weighted using the ENAHO household expansion factor. Food expenditure values are nominal annualized Peruvian soles. Source: ENAHO 2015–2024.

Table S5. Sensitivity analyses using alternative specifications of the available food variety outcome

| Variable | Category | ≤64 products, aPR (95% CI) | p-value | Continuous score, adjusted mean difference (95% CI) | p-value |
| --- | --- | --- | --- | --- | --- |
| Survey period | 2015–2019 | Reference | — | Reference | — |
|  | 2020–2021 | **0.89 (0.87–0.91)** | <0.001 | **1.04 (0.66 to 1.43)** | <0.001 |
|  | 2022–2024 | **1.30 (1.27–1.32)** | <0.001 | **−7.76 (−8.09 to −7.42)** | <0.001 |
| Area of residence | Urban | Reference | — | Reference | — |
|  | Rural | **1.13 (1.11–1.15)** | <0.001 | **−2.83 (−3.15 to −2.52)** | <0.001 |
| Natural region | Coast | Reference | — | Reference | — |
|  | Highlands | **1.21 (1.17–1.24)** | <0.001 | **−4.48 (−5.01 to −3.96)** | <0.001 |
|  | Rainforest | **1.51 (1.44–1.58)** | <0.001 | **−9.33 (−10.17 to −8.50)** | <0.001 |
|  | Metropolitan Lima | **1.07 (1.03–1.12)** | 0.001 | **−1.40 (−2.14 to −0.66)** | <0.001 |
| Poverty status | Non-poor | Reference | — | Reference | — |
|  | Non-extreme poor | **1.12 (1.10–1.14)** | <0.001 | **−0.96 (−1.29 to −0.64)** | <0.001 |
|  | Extreme poor | **1.66 (1.62–1.71)** | <0.001 | **−12.26 (−12.85 to −11.68)** | <0.001 |
| Household size | 1–2 members | Reference | — | Reference | — |
|  | 3–4 members | **0.35 (0.35–0.36)** | <0.001 | **24.15 (23.87 to 24.43)** | <0.001 |
|  | ≥5 members | **0.21 (0.21–0.22)** | <0.001 | **35.71 (35.35 to 36.07)** | <0.001 |
| Children younger than 5 years | No | Reference | — | Reference | — |
|  | Yes | **0.87 (0.85–0.90)** | <0.001 | **3.46 (3.16 to 3.76)** | <0.001 |
| Sex of household head | Male | Reference | — | Reference | — |
|  | Female | **0.92 (0.90–0.93)** | <0.001 | **2.15 (1.90 to 2.39)** | <0.001 |
| Age of household head | 18–39 years | Reference | — | Reference | — |
|  | 40–59 years | **0.87 (0.85–0.89)** | <0.001 | **2.98 (2.68 to 3.27)** | <0.001 |
|  | ≥60 years | **0.85 (0.84–0.87)** | <0.001 | **3.36 (3.02 to 3.71)** | <0.001 |
| Educational level of household head | Higher | Reference | — | Reference | — |
|  | Secondary | **0.94 (0.92–0.96)** | <0.001 | **0.85 (0.54 to 1.16)** | <0.001 |
|  | Primary | **0.98 (0.96–1.00)** | 0.078 | **0.10 (−0.25 to 0.44)** | 0.586 |
|  | No formal/initial | **1.09 (1.05–1.12)** | <0.001 | **−2.72 (−3.25 to −2.20)** | <0.001 |
| Food expenditure per capita | Highest tertile | Reference | — | Reference | — |
|  | Middle tertile | **1.75 (1.71–1.79)** | <0.001 | **−15.12 (−15.41 to −14.83)** | <0.001 |
|  | Lowest tertile | **4.04 (3.95–4.14)** | <0.001 | **−35.30 (−35.65 to −34.94)** | <0.001 |

Note: aPR, adjusted prevalence ratio; CI, confidence interval. The binary sensitivity analysis defined low available food variety using the pooled survey-weighted 25th percentile (≤64 distinct food products). The continuous analysis modeled the available food variety score without dichotomization; positive adjusted mean differences indicate a higher number of available food products and negative values indicate a lower number relative to the reference category. Both sensitivity models used the same covariate structure as Model 2, including survey period, area of residence, natural region, poverty status, household size, presence of children younger than five years, sex, age and educational level of the household head, per-capita household food expenditure tertile, and department fixed effects. The analyses incorporated the ENAHO complex survey design and used complete-case analysis (n = 340,239).

Table S6. Sensitivity analysis restricted to food-product codes observed across all ENAHO survey years, 2015–2024

| Survey period | Weighted prevalence (%) | Adjusted PR (95% CI) | p-value |
| --- | --- | --- | --- |
| 2015–2019 | 33.9 | Reference | — |
| 2020–2021 | 29.5 | 0.93 (0.91–0.95) | <0.001 |
| 2022–2024 | 38.0 | 1.28 (1.26–1.30) | <0.001 |

Note: The available food variety score was reconstructed using only the 463 food-product codes observed in every annual analytic file from 2015 through 2024. Low available food variety was defined using the same fixed threshold of ≤71 products as in the primary analysis. PR, prevalence ratio; CI, confidence interval. The adjusted modified Poisson model used the same covariate structure as Model 2, including survey period, area of residence, natural region, poverty status, household size, presence of children younger than five years, sex, age and educational level of the household head, per-capita household food expenditure tertile, and department fixed effects. The model incorporated the ENAHO complex survey design and used complete-case analysis (n = 340,239).

Table S7. Model 1: adjusted associations with low available food variety before adjustment for per-capita household food expenditure, ENAHO 2015–2024

| Associated factor | Model 1 aPR (95% CI) | p-value |
| --- | --- | --- |
| Survey period |  |  |
| 2015–2019 | Reference | — |
| 2020–2021 | 0.83 (0.81–0.85) | <0.001 |
| 2022–2024 | 1.03 (1.02–1.05) | <0.001 |
| Area of residence |  |  |
| Urban | Reference | — |
| Rural | 1.11 (1.09–1.13) | <0.001 |
| Natural region |  |  |
| Coast | Reference | — |
| Highlands | 1.26 (1.23–1.29) | <0.001 |
| Rainforest | 1.45 (1.39–1.51) | <0.001 |
| Metropolitan Lima | 1.01 (0.97–1.05) | 0.575 |
| Poverty status |  |  |
| Non-poor | Reference | — |
| Non-extreme poor | 1.80 (1.77–1.82) | <0.001 |
| Extreme poor | 2.72 (2.67–2.78) | <0.001 |
| Household size |  |  |
| 1–2 members | Reference | — |
| 3–4 members | 0.46 (0.45–0.46) | <0.001 |
| ≥5 members | 0.33 (0.32–0.34) | <0.001 |
| Children younger than 5 years |  |  |
| No children <5 years | Reference | — |
| At least one child <5 years | 0.87 (0.85–0.88) | <0.001 |
| Sex of household head |  |  |
| Male | Reference | — |
| Female | 0.91 (0.90–0.92) | <0.001 |
| Age of household head |  |  |
| 18–39 years | Reference | — |
| 40–59 years | 0.86 (0.85–0.88) | <0.001 |
| ≥60 years | 0.83 (0.81–0.84) | <0.001 |
| Educational level of household head |  |  |
| Higher | Reference | — |
| Secondary | 1.05 (1.03–1.07) | <0.001 |
| Primary | 1.11 (1.09–1.13) | <0.001 |
| No formal/initial | 1.22 (1.19–1.26) | <0.001 |

Note: aPR, adjusted prevalence ratio; CI, confidence interval. Model 1 was estimated using modified Poisson regression with robust variance and adjusted for survey period, area of residence, natural region, poverty status, household size, presence of children younger than five years, sex, age and educational level of the household head, and department fixed effects. Per-capita household food expenditure was not included in Model 1. The analysis incorporated ENAHO sampling weights, strata, and clustered primary sampling units. Complete-case analysis was used (n = 340,239).
